# Detection of prostate cancer with ^18^F-DCFPyL PET/CT compared to final histopathology of radical prostatectomy specimens: is PSMA-targeted biopsy feasible? The DeTeCT trial

**DOI:** 10.1007/s00345-020-03490-8

**Published:** 2020-10-20

**Authors:** Y. J. L. Bodar, B. H. E. Jansen, J. P. van der Voorn, G. J. C. Zwezerijnen, D. Meijer, J. A. Nieuwenhuijzen, R. Boellaard, N. H. Hendrikse, O. S. Hoekstra, R. J. A. van Moorselaar, D. E. Oprea-Lager, A. N. Vis

**Affiliations:** 1 Department of Urology, Amsterdam University Medical Centres (VU University), De Boelelaan 1117, 1081 HV Amsterdam, The Netherlands; 2 Department of Radiology and Nuclear Medicine, Amsterdam University Medical Centres (VU University), Amsterdam, The Netherlands; 3 Prostate Cancer Network, Amsterdam, The Netherlands; 4 Department of Pathology, Amsterdam University Medical Centres (VU University), Amsterdam, The Netherlands; 5 Department of Clinical Pharmacology and Pharmacy, Amsterdam University Medical Centres (VU University), Amsterdam, The Netherlands

**Keywords:** ^18^F-DCFPyL PET/CT, Primary detection, Prostate cancer, PSMA, Targeted biopsy

## Abstract

**Purpose:**

In primary prostate cancer (PCa) patients, accurate staging and histologic grading are crucial to guide treatment decisions. ^18^F-DCFPyL (PSMA)-PET/CT has been successfully introduced for (re)staging PCa, showing high accuracy to localise PCa in lymph nodes and/or osseous structures. The diagnostic performance of ^18^F-DCFPyL-PET/CT in localizing primary PCa within the prostate gland was assessed, allowing for PSMA-guided targeted-prostate biopsy.

**Methods:**

Thirty patients with intermediate-/high-risk primary PCa were prospectively enrolled between May 2018 and May 2019 and underwent ^18^F-DCFPyL-PET/CT prior to robot-assisted radical prostatectomy (RARP). Two experienced and blinded nuclear medicine physicians assessed tumour localisation within the prostate gland on PET/CT, using a 12-segment mapping model of the prostate. The same model was used by a uro-pathologist for the RARP specimens. Based on PET/CT imaging, a potential biopsy recommendation was given per patient, based on the size and PET-intensity of the suspected PCa localisations. The biopsy recommendation was correlated to final histopathology in the RARP specimen. Sensitivity, specificity, positive predictive value (PPV) and negative predictive value (NPV) for clinically significant PCa (csPCa, Gleason score ≥ 3 + 4 = 7) were assessed.

**Results:**

The segments recommended for potential targeted biopsy harboured csPCA in 28/30 patients (93%), and covered the highest Gleason score PCa segment in 26/30 patient (87%). Overall, 122 of 420 segments (29.0%) contained csPCa at final histopathological examination. Sensitivity, specificity, PPV and NPV for csPCa per segment using ^18^F-DCFPyL-PET/CT were 61.4%, 88.3%, 68.1% and 84.8%, respectively.

**Conclusions:**

When comparing the PCa-localisation on ^18^F-DCFPyL-PET/CT with the RARP specimens, an accurate per-patient detection (93%) and localisation of csPCa was found. Thus, ^18^F-DCFPyL-PET/CT potentially allows for accurate PSMA-targeted biopsy.

**Electronic supplementary material:**

The online version of this article (10.1007/s00345-020-03490-8) contains supplementary material, which is available to authorized users.

## Introduction

Prostate cancer (PCa) is the most common cancer in men in the Western world [1, 2]. Histopathological verification is required to confirm the diagnosis and is standardly attained through ultra-sound-guided systematic prostate biopsies [3]. These random biopsies are subject to sampling error, however, resulting in false-negative outcomes and imprecise tumour-risk assessment [4, 5]. To overcome this, multi-parametric magnetic resonance imaging (mpMRI) prior to systematic biopsy has been implemented in clinical guidelines, enabling targeted biopsies of radiologically suspected lesions (MRI-TBx) [3, 6–8].

Besides conventional imaging modalities such as mpMRI, novel imaging techniques including prostate-specific membrane antigen-positron emission tomography/computed tomography (PSMA-PET/CT) have been introduced. PSMA is significantly overexpressed in malignant prostate cells, correlates with higher tumour grades and represents a marker of tumour aggressiveness [9, 10]. PSMA-PET/CT-imaging has been shown to accurately identify the primary prostate tumours, with detection rates of 98–100% [11, 12]. PSMA-PET/CT could thus be used to localize and guide targeted prostate biopsy in patients with clinically suspected PCa. Furthermore, PSMA-PET/CT would simultaneously provide screening for bone and lymph-node metastases, as it is repeatedly found to be more sensitive than conventional imaging (i.e., MRI, bone scintigraphy and CT) in the initial staging setting [13, 14].

This is the first prospective study on the accuracy of ^18^F-DCFPyL (PSMA) PET/CT imaging for the primary detection of PCa. The primary aim was to assess the accuracy of ^18^F-DCFPyL-PET/CT to localise primary PCa within the prostate gland, by comparing imaging results from ^18^F-DCFPyL-PET/CT to final histopathology of the robot-assisted radical prostatectomy (RARP) specimen. The secondary objectives were to investigate the ability of ^18^F-DCFPyL-PET/CT to provide a recommendation for potential targeted biopsy and to assess the diagnostic accuracy of determining local tumour stage (pT).

## Methods

### Study design and patient population

This was a prospective, non-randomised study in patients with diagnosed primary PCa. Pre-operative imaging results were compared to histopathology following RARP. All subjects signed informed-consent for the collection of their clinical data. The study has been approved by the ethical review board of the Amsterdam University Medical Centre (AUMC) (review number 2017.543). Patients were enrolled consecutively between May 2018-May 2019 in Amsterdam UMC, location VUmc.

Patients had histologically proven, intermediate or high-risk, PCa, for which they underwent RARP [3, 15]. Of all included patients, age, prostate volume, initial prostate-specific antigen (PSA)-level, clinical T-stage, pathological biopsy features (histopathological grade, number of cores with cancer) and European Association of Urology (EAU)-risk category were collected [3, 15]. A 12-segment anatomic mapping model of the prostate was used to localise and characterise the prostatic tumours, with 2 additional segments representing the seminal vesicles (pT3b) (Appendix-1 in ESM) [16].

### Imaging protocol

Patients were staged with ^18^F-DCFPyL which was synthesised under Good Manufacturing Practices conditions, as described by Jansen et al. [17, 18]. PET images were acquired at a median of 118 min after injection of the radiotracer (interquartile range [IQR] 113–122 min) with a median dose of 313 MBq ^18^F-DCFPyL (IQR 299–324 MBq), and a median of 5.4 weeks (IQR 3.0–7.2) prior to surgery. Image-acquisitions were performed using a Philips Ingenuity TF (Philips Healthcare®, NL/USA)-PET/CT system. No diuretics were administered prior to the scan. The scan trajectory included mid-thighs to skull-base, with 4 min per bed position. All PET scans were combined with a diagnostic CT scan (110 mAs, 120 kV), without contrast-enhancement. Images were corrected for decay, scatter, random coincidences, and photon attenuation.

Images were reconstructed with a BLOB-based Ordered-Subsets Expectations Maximization algorithm (Philips, 3 iterations; 33 subsets) [19]). The default Ordered-Subsets Expectations Maximization with Time-of-Flight reconstruction was used. Reconstructions included both 4 mm for semi-quantification purposes, and 2 mm slices for visual interpretation (matrix size 144 × 144, slice thickness 4 mm; matrix size 288 × 288, slice thickness 2 mm).

### Image interpretation and ^18^F-DCFPyL-based potential biopsy recommendation

Scan interpretation was performed blinded for the pathology results and other imaging by two nuclear medicine physicians (DO,GZ) with ample experience in ^18^F-DCFPyL-PET/CT reading (> 300 scans), in consensus. The readers used the 12-segment mapping model to demarcate the image-detected tumour extent (Appendix-1 in ESM) [20]. For all positive segments, the readers’ diagnostic confidence was evaluated using a five-point scale, alike the PSMA-RADS classification [21] (score 1–2 ‘benign’; 3 ‘equivocal’; 4–5 ‘likely PCa’). PSMA-RADS 4–5 were defined as suspicious for PCa (‘positive’), and were used for the final diagnostic accuracy analysis. Based on PET/CT-imaging, two segments per patient were indicated to be potentially targeted by prostate biopsy. These segments were selected based on both visual interpretation (location, size) and semi-quantification by determining the highest standardised uptake values (SUV_max_) of the suspected lesions. Finally, the readers indicated if radiological extra-capsular extension (ECE, rT-stage 3a) or invasion into the seminal vesicles (rT3b) was suspected.

### Pathology analysis

RARP specimens were processed according to clinical routine and the International Society of Urological Pathology (ISUP) guidelines [22]. All specimens were fixated in formaldehyde (10%) directly after surgery. The surface of the specimens was inked and the apex and base (bladder neck) were removed. The mid part of the specimen was cut perpendicular to the urethra in 4 mm slices. The apex and base parts were cut in a sagittal fashion. Histologic slices were produced after sectioning in quadrants. Blinded by PET/CT results, an experienced uro-pathologist (PV) reviewed all slices and delineated all tumour depositions on the 12-segment mapping model of the prostate and the 2 segments of the seminal vesicles (Appendix-1 in ESM). For all segments that contained tumour, a Gleason score and ISUP grade was provided and the presence of ECE (pT3a). Clinically significant PCa (csPCa) was defined as PCa with Gleason score ≥ 3 + 4 = 7 (ISUP grade ≥ 2) or any tumour with ≥ pT3a. The index lesion was defined as the largest lesion with the highest ISUP grade or stage.

### Statistical analysis

The localisation of the detected prostate tumour by ^18^F-DCFPyL-PET/CT was matched to the histopathology results and the sensitivity, specificity, positive predicting value (PPV), and negative predicting value (NPV) were calculated on a segment basis. Correlation of ^18^F-DCFPyL-PET/CT with histopathology was considered if exactly the same segment was demarcated (total agreement). Since there are no anatomical landmarks to delineate the segments, artificial segmentation can occur, causing a mismatch between the PET/CT- and histopathological findings while both correspond with the same lesion. Therefore, a second analysis of diagnostic accuracy was performed, in which PET correlation was also considered if there was a discrepancy of up to 1 region in the coronal or sagittal plane (near-total agreement) [23, 24]. Reciever‐operating characteristic curves (ROC) and area under the curve (AUC) analysis were performed to explore the accuracy of PSMA-PET/CT in the detection of segments containing csPCa based on the 5-point scale. For the assessment of pathological tumour stage (pT), we investigated the accuracy of ^18^F-DCFPyL-PET/CT to differentiate locally advanced disease (> rT3a) from prostate-confined disease (rT2). Numerical variables were summarised with median values and interquartile ranges (IQR); categorical variables with proportions (%). To compare medians of non-parametric data, the Mann–Whitney-Wilcoxon test and the Kruskal-Wallis test were used (significance set at *p* < 0.05). Statistical analysis was performed with IBM® SPSS® Statistics for Windows®, version 26.

## Results

### Patient characteristics

A total of 30 patients was included in this study, having a median initial PSA-level of 11.1 ng/ml (IQR 5.8–22.4). According to EAU guidelines, 10/30 (33.3%) patients had intermediate-risk PCa and 20/30 (66.6%) had high-risk PCa [15]. Pre-operative and post-operative characteristics of included patients are listed in Table 1.

**Table 1:** Pre- and postoperative characteristics of patients undergoing ^18^F-DCFPyL-PET/CT before robot-assisted radical prostatectomy

Baseline (pre-operative) characteristics		
	Median	IQR
Age (years)	68.5	64.3–69.8
Prostate volume (ml)	44.0	32.2–69.0
Initial PSA (ng/ml)	11.1	5.8–22.4
Positive biopsy cores (% of total cores)	50.0	35.0–71.0
MSKCC risk of lymph-node metastases (%)	13.4	10.6–28.0
	*n*	%
Biopsy ISUP grade^a^		
1	0	0.0
2	9	30.0
3	7	23.3
4	11	36.7
5	3	10.0
Total	30	100.0
Clinical T stage		
1c	5	16.6
2a/b	18	60.0
2c	6	20.0
3a	1	3.3
Total	30	100.0
EAU risk category		
Intermediate	10	33.3
High	20	66.6
Total	30	100.0

Pathology (post-operative) results		
ISUP category^**a**^		
1	0	0.0
2	13	43.3
3	6	20.0
4	2	6.7
5	6	20.0
Total	30	100.0
Pathological tumour (pT) stage		
pT2	16	53.3
pT3a	10	33.3
pT3b	4	13.3
Total	30	100.0
Pathological Lymph-node (N) stage		
0	29	96.7
1	1	3.3
Total	30	100.0

*IQR* interquartile range, *PSA* prostate-specific antigen, *MSKCC* Memorial Sloan Kettering Cancer Centre, *ISUP* International Society of Urological Pathology, *EAU* European Association of Urology

^a^ISUP definition

ISUP 1 = Gleason score 3 + 3 = 6

ISUP 2 = Gleason score 3 + 4 = 7

ISUP 3 = Gleason score 4 + 3 = 7

ISUP 4 = Gleason score 4 + 4 = 8/Gleason score 3 + 5 = 8/Gleason score 5 + 3 = 8

ISUP 5 = Gleason score 4 + 5 = 9/Gleason score 5 + 4 = 9/Gleason score 5 + 5 = 10

### Accuracy of ^18^F-DCFPyL-PET/CT to detect local prostate cancer on a segmental level

All patients showed PSMA expression in the prostate. In 30 evaluated patients, 420 segments (12 prostate segments + 2 seminal vesicle segments per patient) could be used both for PET/CT and histopathological mapping evaluation. PCa was present in 129 of the 420 (30.7%) segments on histopathological examination, and csPCa was found in 122 of the 420 segments (29.0%) (median 3 segments per patient, IQR 2–5). The sensitivity, specificity, PPV and NPV of ^18^F-DCFPyL-PET/CT to detect csPCa per segment with total agreement was 61.4% (95%CI 52.2–70.0%), 88.3% (95%CI 83.9–91.6%), 68.1% (95%CI 58.5–76.6%), and 84.8% (95%CI 80.2–88.5%), respectively (Appendix-2 in ESM). For near-total agreement, the sensitivity, specificity, PPV and NPV of ^18^F-DCFPyL PET/CT to detect csPCa per segment was 84.4% (95%CI 76.5–90.1%), 97.0% (95%CI 94.1–98.5%), 92.0% (95%CI 84.9–96.0%), and 93.8% (95%CI 90.3–96.1%), respectively. The area under the curve (AUC) of ^18^F-DCFPyL-PET/CT was 0.78 (95%CI 0.73–0.84) for the total agreement scores, and 0.85 (95%CI 0.80–0.90) for the near-total agreement scores (Appendix-3 in ESM). True positive segments had a median SUV_max_ of 8.26 (IQR 5.25–11.40), which was significantly higher than the median SUV_max_ of false-positive segments of 4.06 (IQR 3.56–5.10) (*p* = 0.02). The median SUV_max_ of true positive segments did not correlate with ISUP grade groups (*p* = 0.95) (Appendix-4 in ESM).

### Potential targeted biopsy recommendation on a patient level

**Fig. 1:**
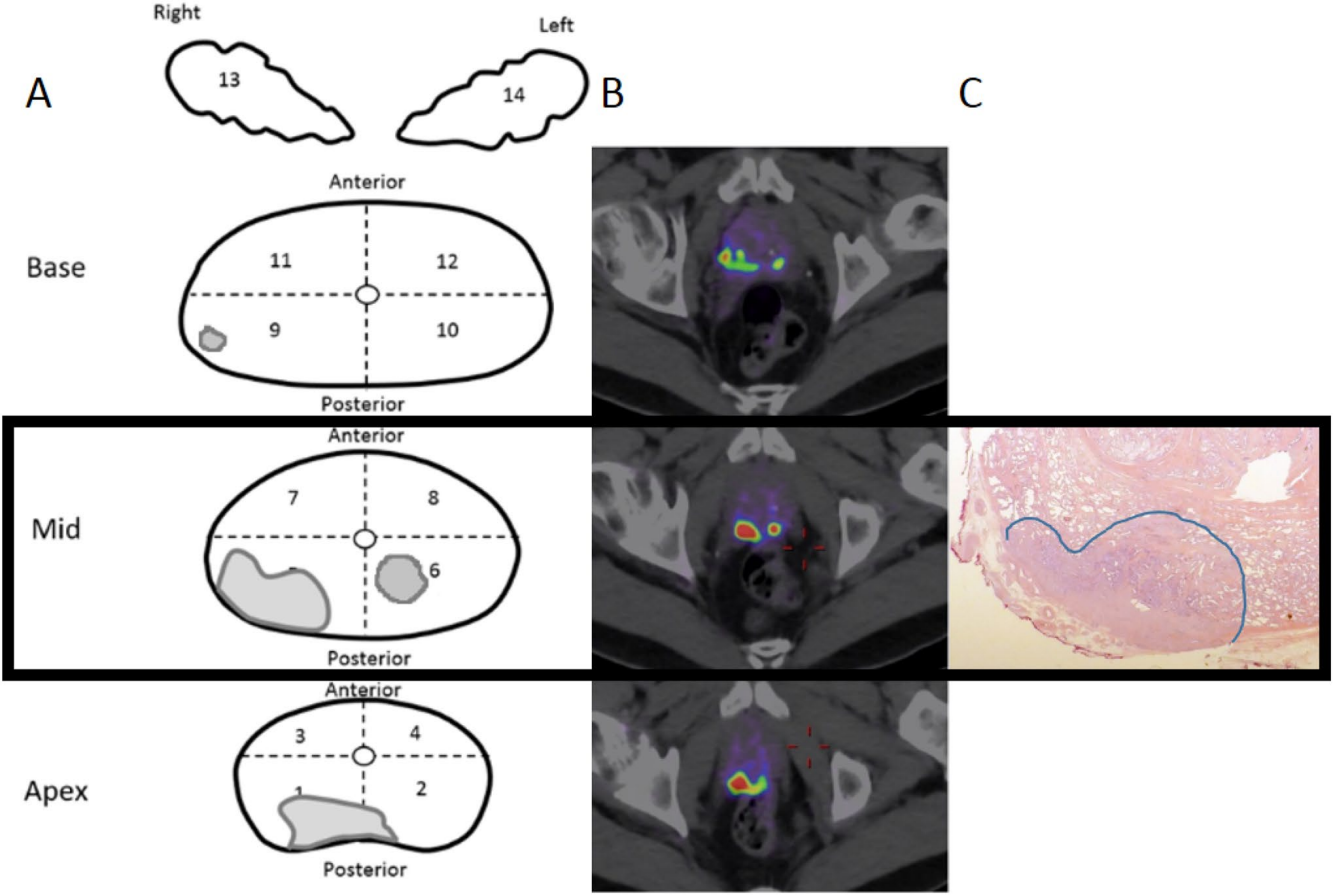
67-Year-old man with cT3a, Gleason score 3 + 4 = 7 prostate cancer and an initial PSA 27 ng/ml considered candidate for radical prostatectomy with extended pelvic lymph-node dissection. **a** Schematic drawing of the 12 prostate segments included in the study. **b** Transversal fused ^18^F-DCFPyL-PET and CT show intense focal uptake in the right posterior midgland and apex segments, with extracapsular extension. Based on the highest SUV_max_ of 6.09 and tumour size, segment 5 is recommended for potential targeted biopsy. **c** histopathology (hematoxylin and eosin stain, original magnification × 10) shows a tumour focus, in both segment 5 and 1 with a Gleason score 3 + 4 = 7 prostate cancer with extraprostatic extension (pT3a), hereby confirming the index lesion localisation by PET

The primary potential biopsy recommendation by the nuclear medicine physician harboured csPCa in 24/30 (80.0%) patients and detected the index PCa lesion in 23/30 (76.6%) patients. When both the primary and secondary recommended segments would potentially be targeted, ^18^F-DCFPyL-PET/CT revealed csPCa in 28/30 (93.3%) patients. Moreover, it pinned the index PCa lesion in 26/30 (86.7%) patients. An example of potential ^18^F-DCFPyL**-**guided biopsy recommendation and concurrent histopathological examination of the RARP specimen is shown in Fig. 1. Potential biopsy recommendation that matched the index PCa lesion had a median SUV_max_ 8.62 (IQR 6.41–12.62). The recommendation for potential biopsy from the nuclear physicians that matched csPCA had a median SUV_max_ of 8.55 (IQR 6.34–13.79), and was significantly higher than the recommended potential biopsy segments that did not contain csPCa (median SUV_max_ of 3.10 (IQR 2.86–3.87) (*p* = 0.02).

### Local staging

Final histopathological analysis revealed pT3a in 10/30 (33.3%) patients, and pT3b in 4/30 (13.3%) patients. The sensitivity, specificity, PPV and NPV of ^18^F-DCFPyL-PET/CT to detect locally advanced tumour growth (≥ pT3a) was 35.7% (95%CI 14.0–64.3%), 93.8% (95%CI 67.7–99.7%), 83.3% (95%CI 36.5–99.1%), and 62.5% (95%CI 40.8–80.4%), respectively (Appendix-5 in ESM). For the detection of pT3a sub-stage, the sensitivity, specificity, PPV and NPV of ^18^F-DCFPyL-PET/CT were 20.0% (95%CI 3.5–55.8), 100% (95%CI 80.0–100), 100.0% (95%CI 19.7–100), and 71.4% (95%CI 51.1–86.0), respectively. For the detection of pT3b sub-stage, the sensitivity, specificity, PPV and NPV of ^18^F-DCFPyL-PET/CT were 75.0% (95%CI 21.9–98.7), 92.3% (95%CI 73.4–98.7), 60.0% (95%CI 17.0–92.7), and 96.0% (95%CI 77.7–99.8), respectively.

## Discussion

This is the first prospective study in which ^18^F-DCFPyL-PET/CT imaging was used to locate primary PCa within the prostate gland, exploring the diagnostic potential of PSMA-based targeted biopsies. A total of 30 patients diagnosed with intermediate and high-risk PCa that underwent ^18^F-DCFPyL-PET/CT prior to RARP was analysed. When using a prostate-mapping model, the potential ^18^F-DCFPyL-PET/CT-based targeted biopsy recommendation detected csPCa in 28/30 (93.3%) patients. Moreover, it detected the index PCa lesion in 26/30 (86.7%) patients. Potentially, this makes PSMA-targeted biopsy a diagnostic tool that may adequately guide precision prostate biopsy. In biopsy-naive patients at increased risk of metastatic spread, and in whom staging imaging is mandatory (e.g. PSA ≥ 20), ^18^F-DCFPyL-PET/CT could potentially be used simultaneously to stage patients and to target PSMA-avid prostatic lesions suspicious for PCa.

^18^F-DCFPyL-PET/CT imaging demonstrated a moderate per segment-based sensitivity for the detection of csPCa of 61.4%, at a 88.3% specificity. The moderate sensitivity indicates that ^18^F-DCFPyL-PET/CT was not able to detect all localised csPCa. Segmentation of the prostate gland is problematic, as no clear anatomical landmarks are available to delineate the different segments within the prostate (Appendix-1 in ESM). A tumour located on the border of the apex and middle part of the prostate could be classified in different segments by the nuclear medicine physician and uro-pathologist, while in fact, they detected the same lesion. Therefore, the near-agreement score was introduced to approximate clinical reality. The sensitivity of the near-agreement score of ^18^F-DCFPyL-PET/CT imaging for the detection of csPCa was higher at 84.4% with a specificity of 97.0%.

MpMRI has found a prominent place in the identification and localisation of PCa [8]. Few studies have directly compared the outcome of mpMRI to that of PSMA-PET/CT for the detection of localised PCa. Scheltema et al. analysed 56 patients with intermediate-risk PCa who underwent ^68^Ga-PSMA-PET/CT and mpMRI prior to RARP [20]. This study used the same 12-segment-based model of the prostate, and a leniency method similar to our near-agreement score. The patient-based sensitivity for detecting ISUP grade 2–3 PCa was 100% for PSMA-PET/CT vs 98% for mpMRI (PI-RADS 3–5). The segment-based sensitivity and specificity for PSMA-PET/CT was 88% (95% CI 83–92) and 93% (95% CI 91–95) compared 68% (95% CI 61–75) and 91% (95% CI 87–93) for mpMRI (PI-RADS 3–5).

In another study, Kesch et al. [23] studied 10 patients with primary high-risk PCa who underwent ^18^F- PSMA-1007-PET/CT and mpMRI with subsequent RARP. Nine of the men were diagnosed with MRI-TBx and 1 with systematic biopsy. Using a 36-segment mapping model, a similar assessment of agreement and near-total agreement was used. In 10 patients, ^18^F-PSMA-1007 PET/CT showed a high sensitivity (71% for total and 93% for near-total agreement), specificity (81% for total and 92% for near-total agreement), and accuracy (75% for total and 93% for near-total agreement) for the detection of csPCa. Although the specificity was similar, this study did however show lower sensitivity for PSMA-PET/CT compared to mpMRI (86% for total and 92% for near-total agreement). Above mentioned studies implicate that PSMA-PET/CT imaging performs at least equal to mpMRI to locate primary PCa. The rates of mpMRI might have been overestimated as at least a part of included patients in previously mentioned studies were diagnosed by MRI-TBx. Thus, selection bias may have been introduced.

There is therapeutic importance to distinguish between T2 and T3 disease (i.e. for planning nerve-sparing surgery, to opt for active surveillance) [3]. A moderate sensitivity for the detection of pT3a-b of 35.7% was observed using ^18^F-DCFPyL-PET/CT. So, similar to mpMRI, a substantial number of patients with ≥ pT3a was understaged by PSMA-based imaging [8]. However, in a majority of patients, a PSMA-PET/CT rT3a-b finding was confirmed after histopathological examination (PPV for pT3a-b disease of 83.3%). Moreover, the promising specificity for pT3a-b using ^18^F-DCFPyL-PET/CT of 93.8% is congruent with similar ^68^ Ga-PSMA studies (specificity > 90% for T3b disease) [12, 25, 26]. Therefore, we recommend nuclear medicine physicians to report on the presence of rT3a-b specifically.

Our study has inherent limitations. Since the PET/CT resolution is confined at 5 mm, limited diagnostic accuracy for small PCa-foci is to be expected. A selection bias has been introduced due to the selection of patients with biopsy-confirmed csPCa. It is thus unclear how ^18^F-DCFPyL-PET/CT performs in a truly biopsy naïve cohort of patients. The present study was set up to evaluate the capability of PSMA-PET/CT to guide targeted prostate biopsy for the detection of csPCa, not with the goal to discriminate between those who should be or should not be biopsied. Unfortunately, not all patients received a pre-operative mpMRI, limiting direct comparison to ^18^F-DCFPyL-PET/CT. Moreover, some of the patients who received a mpMRI had a longer interval between the mpMRI and the PET/CT scans due to the mpMRI being performed at the referring centre. Finally, since the RARP-specimen will always change shape (due to organ slicing and shrinking artefacts) when it is removed from the body, no truly exact anatomical correlation is possible. Therefore, the partial agreement score was used to correct for this pitfall.

## Conclusions

When comparing the localisation of PCa on ^18^F-DCFPyL-PET/CT with the RARP specimen using anatomical mapping, an accurate per-patient localization (93%) of csPCa was found within the prostate. ^18^F-DCFPyL-PET/CT proves promising for PSMA-targeted biopsy and provides a moderate local staging ability.

## Electronic supplementary material

Below is the link to the electronic supplementary material.

Supplementary file1 (JPG 61 kb)

Supplementary file2 (JPG 138 kb)

Supplementary file3 (JPG 79 kb)

Supplementary file4 (JPG 70 kb)

Supplementary file5 (JPG 177 kb)

## Data Availability

Data are available on request to the corresponding author.
