# Supplementary figures and images for: Detection of prostate cancer with 18F-DCFPyL PET/CT compared to final histopathology of radical prostatectomy specimens: is PSMA-targeted biopsy feasible? The DeTeCT trial
Source: World J Urol. 2020 Oct 20;39(7):2439–46. doi: 10.1007/s00345-020-03490-8 (PMC8332599; doi:10.1007/s00345-020-03490-8)

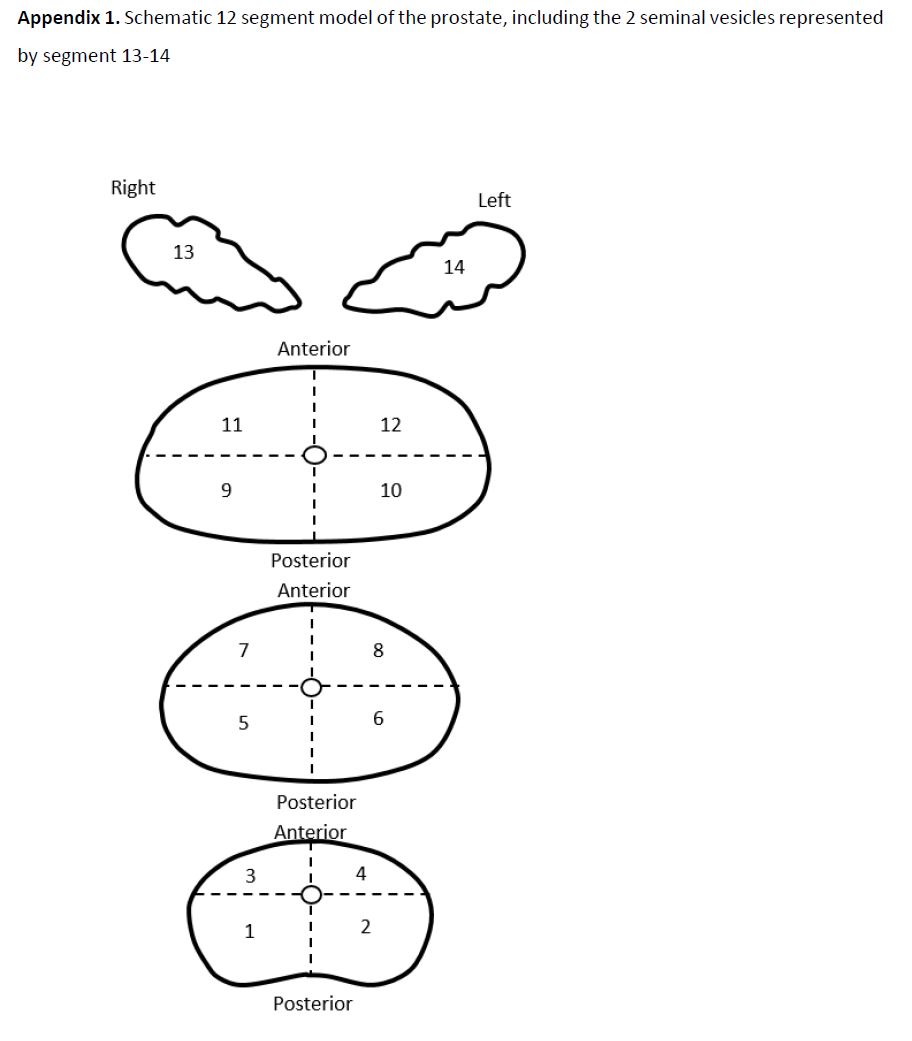

Supplement: Supplementary file 1 — Supplementary file1 (JPG 61 kb) [file 345_2020_3490_MOESM1_ESM.jpg]

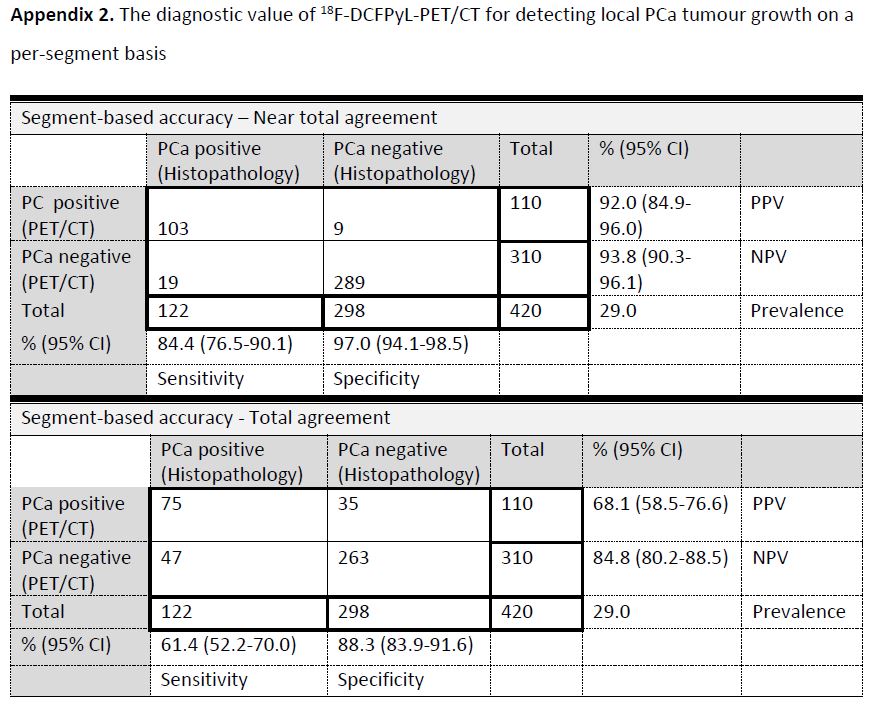

Supplement: Supplementary file 2 — Supplementary file2 (JPG 138 kb) [file 345_2020_3490_MOESM2_ESM.jpg]

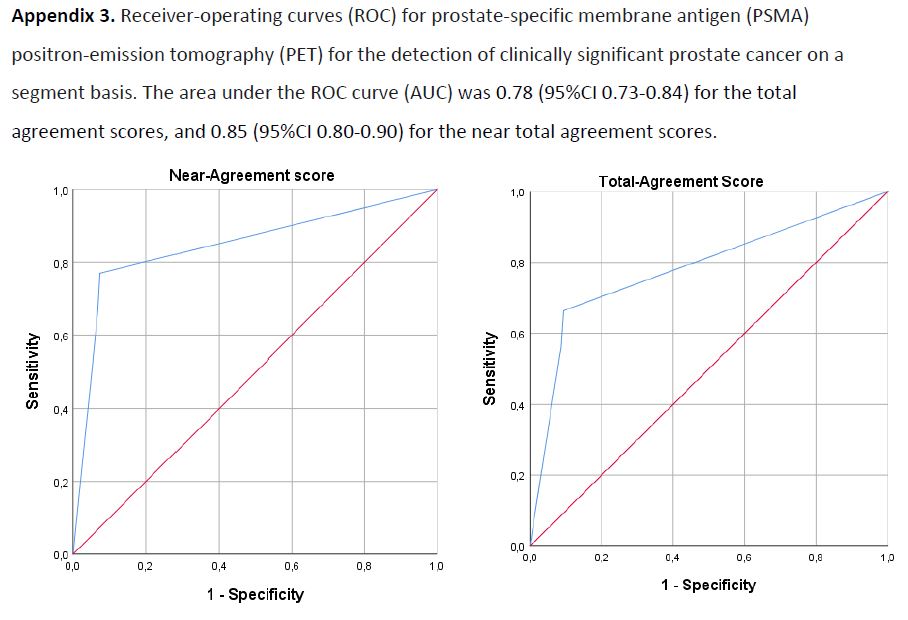

Supplement: Supplementary file 3 — Supplementary file3 (JPG 79 kb) [file 345_2020_3490_MOESM3_ESM.jpg]

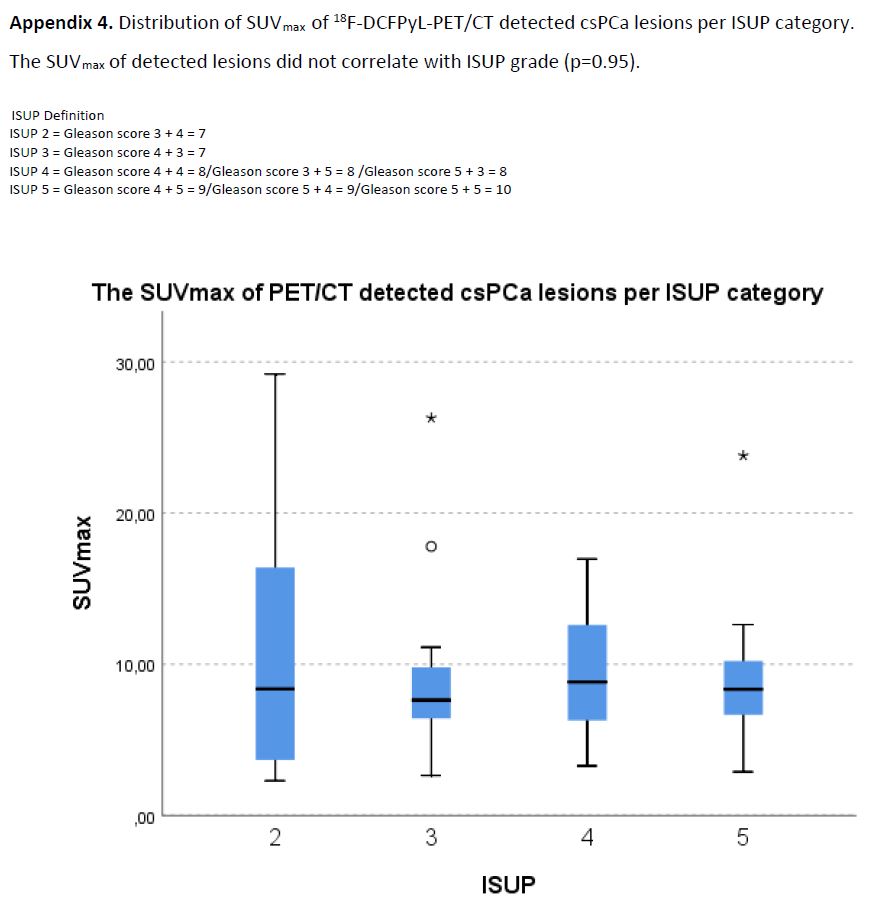

Supplement: Supplementary file 4 — Supplementary file4 (JPG 70 kb) [file 345_2020_3490_MOESM4_ESM.jpg]

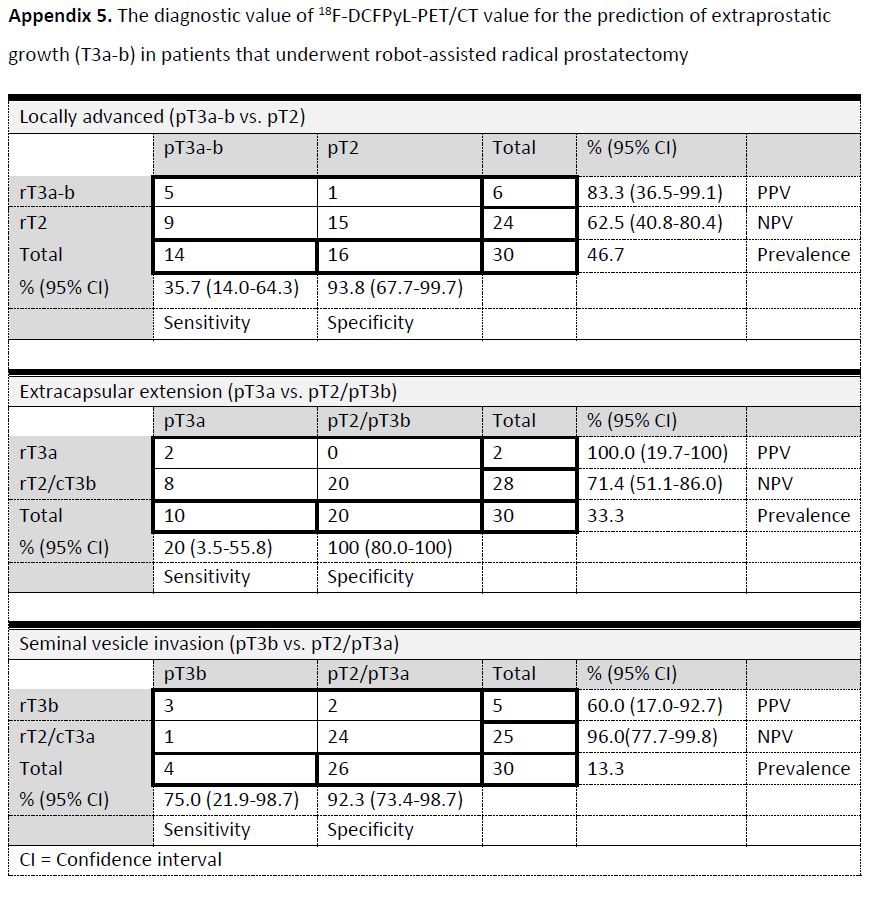

Supplement: Supplementary file 5 — Supplementary file5 (JPG 177 kb) [file 345_2020_3490_MOESM5_ESM.jpg]
